# MicroRNA-182 is a potential biomarker for prognosis of gastric cancer: A protocol for meta-analysis and bioinformatics analysis

**DOI:** 10.1097/MD.0000000000025830

**Published:** 2021-06-04

**Authors:** Dachun Xiao, Jian Xie, Shuai Wu

**Affiliations:** Department of Gastroenterological Surgery, Yongchuan Hospital of Chongqing Medical University, Chongqing, China.

**Keywords:** bioinformatics, gastric cancer, meta-analysis, microRNA-182, protocol

## Abstract

**Background:**

Being the second leading cause of cancer death in the world, gastric cancer is a common malignant tumor in digestive system. Most patients were diagnosed in advanced stage and had poor prognosis. In recent years, related studies have displayed that MicroRNA-182 (miRNA-182) can promote the proliferation, infiltration, metastasis and drug resistance of tumor cells, so it can be used as a new molecular marker for the early diagnosis, prognosis, and treatment of tumors. However, the expression and prognosis of miRNA-182 in gastric cancer are not clear. Therefore, this study conducted a meta-analysis to further clarify the relationship between the expression of miRNA-182 in gastric cancer and prognosis. In addition, a bioinformatics analysis was adopted to further analyze the possible molecular mechanism of miRNA-182, so as to provide a theoretical basis for the diagnosis, treatment, and prognosis of patients suffering from gastric cancer.

**Methods:**

The following electronic databases were searched on computer: Wanfang, Chinese Biomedical Literature Database, Chinese National Knowledge Infrastructure, the Chongqing VIP Chinese Science and Technology Periodical Database, PubMed, Embase, and Web of Science databases. The retrieval time is set to build the database until April 2021. Combined hazard ratios (HRs) and 95% confidence intervals (95% CIs) were used to evaluate the effects of miRNA-182 on the prognosis of gastric cancer. Stata 16.0 software was applied for the meta-analysis. The expression of miRNA-182 in gastric cancer was analyzed by Gene Expression Omnibus database and The Cancer Genome Atlas database. The survival curve of miRNA-182 differential expression was analyzed by OncomiR. The target genes of miRNA-182 were predicted by TargetScan, miRBase, miRTarBase, starBase V2.0, and miRWalk. The target genes were obtained by the intersection of Wayne diagram. DAVID database was used for gene ontology (GO) and Kyoto encyclopedia of genes and genomes enrichment analysis. STRING database and Cytoscape were applied to construct Protein-protein interaction network to obtain key genes (hub gene). The expression of hub gene in gastric cancer was analyzed by gene expression profiling interactive analysis. The survival curve between hub gene and prognosis of gastric cancer was drawn by Kaplan-Meier Plotter database. TIMER database was used to analyze the relationship between hub gene expression and immune cell infiltration in gastric cancer.

**Results:**

The results of this meta-analysis will be submitted to a peer-reviewed journal for publication.

**Conclusion:**

This study provides high-quality evidence support for the expression of miRNA-182 and the prognosis of gastric cancer. Through bioinformatics analysis, we further discussed the mechanism of miRNA-182 in gastric cancer and the understanding of related pathways.

**OSF Registration Number:**

DOI 10.17605/OSF.IO/EHJ6X.

## 1 Introduction

It is reported that, in 2018, there were 1,033,701 new cases of gastric cancer worldwide, with 783,000 of death.^[1]^ Gastric cancer is the third leading cause of cancer-related death after lung and colorectal cancer,^[2]^ because its death accounts for 8.2% of all cancer-related deaths. Due to the lack of early diagnostic markers, most patients with gastric cancer are diagnosed at an advanced stage, and their 5-year survival rate is only 30%.^[3]^ Therefore, an in-depth understanding of the molecular pathogenesis of gastric cancer and the search for biomarkers that are more sensitive to gastric cancer are very important for the improvement of the overall survival (OS) time of patients with gastric cancer.

Micro-RNA (miRNA) is a kind of small molecule RNA, and widely exists in eukaryotic cells. MiRNA can participate in the occurrence and development of tumor, and is closely related to the prognosis of tumor.^[4–7]^ MiRNAs can stably exist in plasma or serum, and may help to clarify the mechanism of tumor growth and find effective markers for early detection of gastric cancer.

MiRNA-182 plays an important role in the key steps of tumorigenesis, such as proliferation, invasion and epithelial-mesenchymal transformation.^[8–11]^ The function of miRNA-182 is complex, and it can inhibit or cause cancer in various cancers. In ovarian cancer, the up-regulated expression of miRNA-182 can promote tumor metastasis.^[8]^ In hepatocellular carcinoma, miRNA-182 promotes tumor metastasis by inhibiting the expression of metastasis inhibitor 1.^[12]^ Some researchers have proposed that miRNA-182 may be used as a biomarker for the diagnosis and prognosis of gastric cancer or as a target for the treatment of gastric cancer.^[13–15]^

Many studies have confirmed that the high expression of miRNA-182 is closely related to the survival of patients with gastric cancer, while the results are uncertain.^[16–18]^ Therefore, this study further analyzes the expression level and prognostic value of miRNA-182 in gastric cancer by carrying out meta-analysis, multi-model database and bioinformatics analysis, and explores its possible molecular mechanism, so as to provide a theoretical basis for the diagnosis, treatment, and prognosis of patients with gastric cancer.

## 2 Methods

### 2.1 Study registration

The protocol of the systematic review has been registered on Open Science Framework. The registration number is DOI 10.17605/OSF.IO/EHJ6X. This meta-analysis protocol is based on the Preferred Reporting Items for Systematic Reviews and Meta-analysis Protocols Statement Guidelines.^[19]^

### 2.2 Data sources and search strategy

The following electronic databases were searched on computer: Wanfang, Chinese Biomedical Literature Database, Chinese National Knowledge Infrastructure, the Chongqing VIP Chinese Science and Technology Periodical Database, PubMed, Embase, and Web of Science databases. The time for literature retrieval is from the establishment of the database to March 2021. The search strategy for PubMed is exhibited in Table 1. The retrieval strategy of other electronic databases is performed on the basis of PubMed. According to the characteristics of each database, the retrieval strategy can be changed slightly.

**Table 1:** Search strategy in PubMed database.

Number	Search terms
#1	Stomach Neoplasms[MeSH]
#2	Cancer of Stomach[Title/Abstract]
#3	Gastric Cancer[Title/Abstract]
#4	Gastric Neoplasms[Title/Abstract]
#5	Stomach Cancer[Title/Abstract]
#6	Cancer of the Stomach[Title/Abstract]
#7	Gastric Cancer, Familial Diffuse[Title/Abstract]
#8	Neoplasms, Gastric[Title/Abstract]
#9	Neoplasms, Stomach[Title/Abstract]
#10	Cancer, Gastric[Title/Abstract]
#11	Cancer, Stomach[Title/Abstract]
#12	Cancers, Gastric[Title/Abstract]
#13	Cancers, Stomach[Title/Abstract]
#14	Gastric Cancers[Title/Abstract]
#15	Gastric Neoplasm[Title/Abstract]
#16	Neoplasm, Gastric[Title/Abstract]
#17	Neoplasm, Stomach[Title/Abstract]
#18	Stomach Cancers[Title/Abstract]
#19	Stomach Neoplasm[Title/Abstract]
#20	or/1–20
#21	MicroRNA-182[Title/Abstract]
#22	miRNA-182[Title/Abstract]
#23	MiR-182[Title/Abstract]
#24	or/21–23
#25	Prognos∗[Title/Abstract]
#26	Survival [Title/Abstract]
#27	or/25–26
#28	#20 and #24 and #27

### 2.3 Inclusion criteria for study selection

Inclusion criteria:

1. All patients were diagnosed with gastric cancer by pathology;
2. Studies evaluated the relationship between high miRNA-182 expression and survival in patients suffering from gastric cancer;
3. The expression level of miRNA-182 in each study was divided into 2 levels based on cut-off value, namely high level and low level.

The exclusion criteria:

1. Case reports, reviews, conference abstracts, and duplicate publications;
2. Animal tests.

### 2.4 Data collection and analysis

#### 2.4.1 Selection of studies

**Figure 1:**
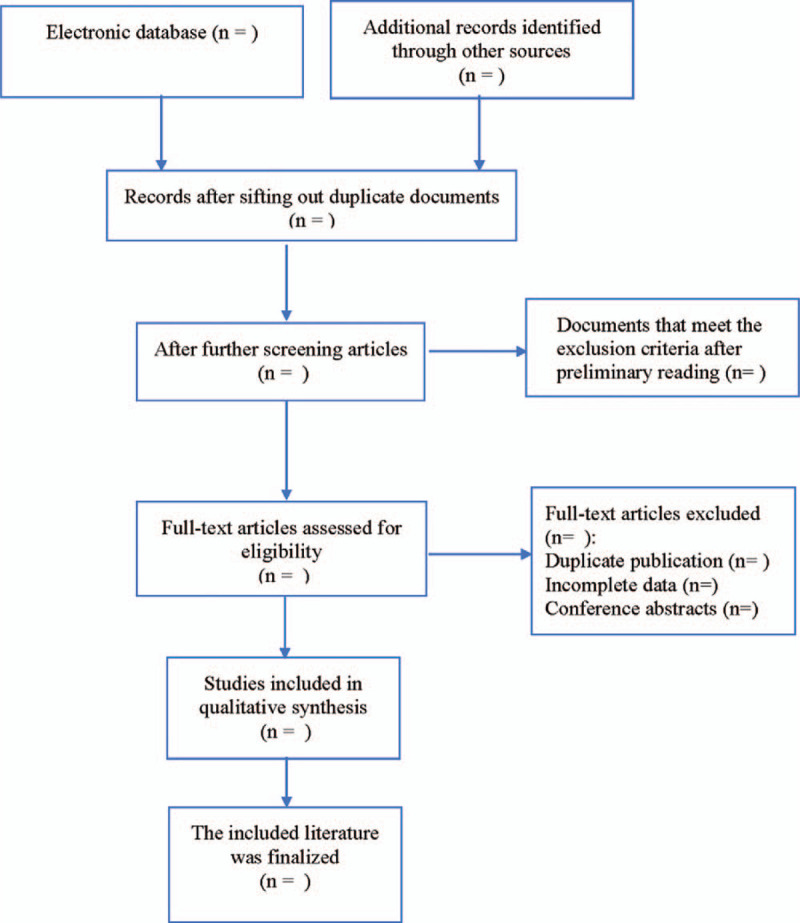
Flow diagram of study selection process.

According to the literature inclusion and exclusion criteria, the retrieval results were screened strictly by 2 researchers, and when there were differences, they were judged by discussion or by a third party. The literature screening process is illustrated in Figure 1.

#### 2.4.2 Data extraction and management

Two reviewers evaluated all the input literatures and analyzed the data independently. In addition, a third reviewer resolved the differences in the event of data inconsistencies. Data extraction contents:

1. The basic characteristics of the study, including the first author, the year of publication, the country of publication, the source of the study population, the number of cases, and detection methods;
2. Extracting directly from the article or using Engauge Digitizer 4.1 software (http://digitizer.sourceforge.net/) to obtain the required data from the survival curve, and through the calculation to obtain the hazard ratio (HR) of OS and disease-free survival, corresponding to the 95% confidence interval (CI), for prognosis analysis;
3. Clinicopathological data, including age, sex, tumor size, TNM stage, differentiation, lymphatic metastasis, and so on.

### 2.5 Assessment of quality in included studies

Literature quality was evaluated based on the Newcastle–Ottawa Quality Assessment Scale.^[20]^ The perfect score is 9 points, and Newcastle–Ottawa Quality Assessment Scale score ≥7 is classified as a high-quality study.^[21]^

### 2.6 Measures of prognosis

OS and disease-free survival will be taken as prognostic outcomes. The results will be expressed as HRs, with 95% CIs.

### 2.7 Management of missing data

If there exists insufficient or missing data in the literature, we would only analyze the currently available data and discuss its potential value.

### 2.8 Statistical analysis

STATA 16.0 (STATA Corporation, College Station, TX) was used for this meta-analysis, and HR and its 95% CIs were used to evaluate the relationship between miRNA-182 expression and clinical prognosis in patients with breast cancer. The Chi-Squared test and *I*^*2*^ values were carried out to assess the heterogeneity among the pooled analysis. When *P* > .1 and *I*^*2*^ < 50%, the fixed-effects model was adopted. By contrast, the random-effects model was adopted when *P* < .1 and *I*^*2*^ > 50%.

### 2.9 Additional analysis

#### 2.9.1 Subgroup analysis

We will conduct a subgroup analysis based on miRNA-182 testing methods, ethnicity, and survival data sources.

#### 2.9.2 Sensitivity analysis

The sensitivity analysis was conducted by adopting the method of excluding the study one by one.

#### 2.9.3 Reporting bias

Egger test was performed to evaluate publication bias, and calculate *t* value and *P* value, with *P* < .05.^[22,23]^ It was considered that there existed publication bias.

### 2.10 Bioinformatics analysis

#### 2.10.1 The expression of miRNA-182

MiRNA-182 expression data of gastric cancer and normal tissues were downloaded from Gene Expression Omnibus database and The Cancer Genome Atlas database. The mean and standard deviation of miRNA-182 expression in gastric cancer tissues and normal controls were calculated by Stata 16.0. In addition, the true positive, true negative, false negative and false positive of each data set were calculated. The area under the curve of the receiver working characteristic curve was established to indicate the diagnostic value of miRNA-182 in gastric cancer.

#### 2.10.2 Expression of miRNA-182 and prognosis of gastric cancer

The survival curve of miRNA-182 differential expression was analyzed by OncomiR online database (http://www.oncomir.org/), and the correlation between miRNA-182 expression and prognosis of gastric cancer patients was discovered.

#### 2.10.3 Target gene prediction

The target genes of miRNA-182 were predicted by TargetScan (http://www.targetscan.org/mamm_31/), miRBase (http://www.mirbase.org/), miRTARBase (http://mirtarbase.cuhk.edu.cn/php/index.php), StarBase V2.0 (http://starbase.sysu.edu.cn/starbase2/index.php), and miRWalk (http://mirwalk.umm.uni-heidelberg.de/). Target genes were obtained by intersecting with Venn diagram.

#### 2.10.4 GO and kyoto encyclopedia of genes and genomes analysis

DAVID database (https://david.ncifcrf.gov/) was used for gene ontology (GO) and Kyoto encyclopedia of genes and genomes enrichment analysis.

#### 2.10.5 Hub gene acquisition

String database (https://www.string-db.org/) and Cytoscape were used to construct Protein-protein interaction network to obtain key genes (hub genes).

#### 2.10.6 The relationship between hub gene expression and prognosis of gastric cancer

The expression of hub gene in gastric cancer was analyzed by gene expression profiling interactive analysis. The survival curves of hub genes and gastric cancer prognosis were plotted by Kaplan–Meier Plotter database (http://www.kmplot.com/analysis/index.php?p=service&cancer=gastric).

#### 2.10.7 Hub gene expression and immune infiltrating cells

TIMER database (http://cistrome.dfci.harvard.edu/TIMER/) was used to analyze the relationship between hub gene expression and immune cell infiltration in gastric cancer.

### 2.11 Ethics

Our research data were derived from published literatures, because there were no patient recruitment and personal information collection. Therefore, ethical approval was not required.

## 3 Discussion

A large number of studies have confirmed that miRNAs is a key molecule in the regulation of post-transcriptional gene expression and plays a key role in the occurrence and development of gastric cancer.^[24]^ Therefore, the in-depth study of the mechanism of microRNA in the occurrence and development of gastric cancer is of great significance for the diagnosis, treatment, and prognosis of gastric cancer.

In most tumors, as an oncogene, miRNA-182 promotes tumor cell proliferation, infiltration, metastasis and the formation of drug resistance.^[25,26]^ MiRNA microarray analysis by Li et al revealed that the expression level of miRNA-182 in intestinal type gastric cancer was 7.43 times higher than that in normal gastric tissue, and the expression level of miRNA-182 was positively correlated with the TNM stage of intestinal type gastric cancer.^[27]^ Yi et al proposed that the expression level was significantly increased in gastric cancer, especially in lymph node metastasis or distant metastasis,^[16]^ which suggests that miRNA-182 can promote the metastasis of tumor cells in gastric cancer.

As a new molecular marker for early diagnosis, prognosis and treatment of tumor, miRNA-182 is very important for the occurrence and development of gastric cancer. In order to investigate the expression of miRNA-182 in gastric cancer and its clinical prognostic significance, we conducted a meta-analysis. At the same time, bioinformatics methods were adopted to analyze the expression of miRNA-182 in gastric cancer and its potential biological processes, so as to provide new ideas and therapeutic targets for further exploration of molecular mechanisms.

## Author contributions

**Conceptualization:** Shuai Wu, Dachun Xiao.

**Data curation:** Shuai Wu, Dachun Xiao.

**Formal analysis:** Dachun Xiao and Jian Xie

**Funding acquisition:** Shuai Wu.

**Methodology:** Dachun Xiao and Jian Xie

**Project administration:** Shuai Wu.

**Resources:** Dachun Xiao.

**Software:** Jian Xie.

**Supervision:** Jian Xie.

**Validation:** Jian Xie.

**Visualization:** Jian Xie.

**Writing – original draft:** Shuai Wu, Dachun Xiao.

**Writing – review & editing:** Shuai Wu, Dachun Xiao.
